# Data for determining the surface properties of carbon fiber in contact interaction with polymeric binders

**DOI:** 10.1016/j.dib.2021.106847

**Published:** 2021-02-08

**Authors:** S.Yu. Voronina, T.A. Shalygina, V.D. Voronchikhin, A.Yu. Vlasov, A.N. Ovchinnikov, N.N. Grotskaya

**Affiliations:** Reshetnev Siberian State University of Science and Technology, Krasnoyarsk, Russia

**Keywords:** Carbonfiber, Fiber surface, Capillary rise, Contact angle

## Abstract

The data presented in this study are the supplementary materials related to the research paper “Determining the Surface Properties of Carbon Fiber in Contact Interaction with Polymeric Binders” (Voronina S.Yu. et al., 2018). The carbon fiber wettability properties before and after heat treatment and the coupling agent IR analysis results are presented. The coupling agent composition affects the fiber wettability and the capillary rise. The polymer binder impregnation rate drives the manufacturing process and the final composite quality. The data would be useful for researchers who study the interphase properties in composites and may help with determining the efficiency of applying certain polymers for wetting carbon fabrics.

## Specification Table

**Table:** 

Subject area	Materials science
Specific subject area	Composites of synthetic fibers
Type of data	Figure Table
How data were acquired	The coupling agent was extracted with toluene from the Torayca T 300 3Kchopped fabric samples and processed with an ultrasonic disperser. The solution was dried in a closed cabinet at 20 °C. Then the dry residue was mortar-milled with 0.15 g of KBr, and formed into tablets under pressure. After that, the tablet spectra were measured. The coupling agent was identified with the Nicolet iS10 FTIR spectrometer and the Smart iTX™ Accessory from Thermo Scientific (USA) with the diamond attenuated total reflectance method (ATR). The coupling agent was removed from the carbon fabric to assess the wettability variation by measuring the capillary rise and the contact angle.
Data format	Raw (Figure) Secondary data: obtained from raw data (Table)
Parameters for data collection	The characteristics of the carbon fibers understudy are the following: filament diameter 7 μm, density 1.76 g cm^−3^, ultimate strength 3530 MPa, elasticity Modulus 230 GPa. Ultra-high resolution scanning electron microscope S-5500 (Hitachi, Japan, 2009) enabled high-resolution imaging. The Nicolet iS10 FTIR spectrometer and the Smart iTX™ Accessory from Thermo Scientific (USA) with the diamond attenuated total reflectance method (ATR) were used for analyzing the carbon fiber extract. The measurements were carried out with a spectral resolution of 4 cm^−1^, averaged over 32 scans, in the range of 4000–400 cm^−1^. The OMNIC software with the application of advanced broken full internal reflection correction was used.
Data source location	Reshetnev Siberian State University of Science and Technology, 31, KrasnoyarskyRabochy *Av*., Krasnoyarsk, Russia 660,037
Data accessibility	Repository name: Mendeley repository https://data.mendeley.com/datasets/729skbm3g5/3
Related research article	Voronina Svetlana Y., Vlasov Anton Y., Voronchikhin Vasylii D., Belov Оleg А., Ivanov Аlexandr V. Determining the Surface Properties of Carbon Fiber in Contact Interaction with Polymeric Binders. Russ. J. ApplChem 91, 1305–1310 (2018). https://doi.org/10.1134/S1070427218080086

## Value of the Data

• Optimization of phase-boundary interactions is one of the main tasks of polymer materials science. Textile coating improves the physical and chemical bonds in the composite. For this reason, the properties of the coupling agent in polymer composites control the efficiency of the interphase adhesive components interaction at their interface surface. The carbon fiber coupling agent affects the binding to the polymer binder and, consequently, the strength of the composite. The strength indicators of products are increasing with the improvement of wettability, since the interphase adhesion between the filler and the binder hinges on the contact angle. Therefore, the data presented in the article as tables and figures are of interest for evaluating the strength of a polymer composite.
• The data would be extremely useful for researchers who study the interphase properties in composites and may help with determining the efficiency of applying certain polymers for wetting carbon fabrics. The data are also usable as reference values for material scientists working with polymer composites.
• The presented contact angle values can be used to estimate the components of the carbon fiber surface energy that characterizes its interphase properties and estimates its compatibility with polymer matrices in polymer composites including shape-memory materials.

## 1 Data Description

Compared to [1], the data presented in this paper contain extra carbon fiber properties: the geometric parameters of the fiber, coating agent content and wetting parameters such as contact angle and capillarity. The data set includes the coupling agent IR and thermal gravimetric analyses results, presented in pictorial form of tables and figures, and provides detailed information about Torayca T 300 3 K brand carbon fiber (Japan). The detailed raw data on determining coating agent properties and mass loss of carbon fiber were deposited at Mendeley public repository as three excel spreadsheets: Table 1, Table 2, Table 3 https://data.mendeley.com/datasets/729skbm3g5/3. Further, these data were processed using the excel office program and presented in form of graphs in Figs. 2 and 3. Fig. 3 shows the processed data on the kinetic curves of wetting in the starting filament samples and filaments after the thermal treatment. Table 4 contains raw data on wetting with water and ethanol before and after carbon fiber heat treatment. Table 5 and Table 6 display raw data set on contact angles in the presence or absence of the coating agent on carbon fiber by using experimental cell for contact angle measurements shown in Fig. 5 and a video editor Virtual Dub, licensed GNU General Public License (GPL). All measurements were performed in triplicates.

**Table 4:** The capillary rise of the carbon fiber filaments under study before and after heat treatment in organic and inorganic solvents.

			Time, min				
Torayca T300 3 K fabric samples	Solvent	Experiment number	0	3	5	10	15
Initial fiber	water	1	0	14	25	29	36
		2	0	16	24	29	35
		3	0	15	26	28	33
fiber after thermal treatment		1	0	27	41	42	45
		2	0	26	38	40	46
		3	0	29	40	39	43
Initial fiber	ethanol	1	0	41	54	62	68
		2	0	39	55	64	68
		3	0	40	54	65	66
fiber after thermal treatment		1	0	34	46	49	55
		2	0	36	47	52	55
		3	0	35	49	54	56

**Table 5:** Initial surface properties of the Torayca T 300 3 K carbon fiber impregnated with the T67 polyurethane resin before heat treatment.

Fractions of a second, ms	Wetting angle, θ, deg.	Drop height, mm	Spot diameter, mm	Spot radius, rad	Surface tension (energy) mN/m	Cos θ	The work of adhesion, mJ/m^2^
0	58.30	7.80	19.80	9.90	40.77	0.53	62.19
40	53.12	6.00	19.90	9.95		0.60	65.23
80	45.00	5.00	20.20	10.00		0.71	69.60
120	42.51	4.20	20.10	10.05		0.74	70.82
160	37.57	4.60	20.80	10.40		0.79	73.08
200	36.53	3.90	20.50	10.25		0.80	73.53
240	32.35	3.50	20.90	10.45		0.84	75.25
280	27.98	3.20	21.20	10.60		0.88	76.77
320	25.82	3.10	21.50	10.75		0.90	77.47
360	25.56	3.00	21.50	10.75		0.90	77.55
400	23.75	2.80	21.50	10.75		0.92	78.09
440	22.00	2.50	21.50	10.75		0.93	78.57
480	15.00	2.10	21.50	10.75		0.97	80.15
520	13.63	1.80	21.50	10.75		0.97	80.39
560	13.50	1.40	19.40	9.70		0.97	80.41
600	13.40	1.30	19.80	9.90		0.97	80.43
640	13.21	1.10	19.80	9.90		0.97	80.46
680	13.10	1.00	19.50	9.75		0.97	80.48
720	13.07	0.90	19.00	9.50		0.97	80.48
760	10.00	0.70	17.30	8.65		0.98	80.92
800	7.13	0.40	17.10	8.55		0.99	81.22
840	2.00	0.30	17.00	8.50		1.00	81.52
880	2.00	0.20	17.00	8.45		1.00	81.52

**Table 6:** Surface properties of the Torayca T 300 3 K carbon fiber impregnated with the T67 polyurethane resin after heat treatment.

Fractions of a second, ms	Wetting angle, θ, deg.	Drop height, mm	Spot diameter, mm	Spot radius, rad	Surface tension (energy) mN/m	Cos θ	The work of adhesion, mJ/m^2^
0	59.04	4.80	12.70	6.35	40.77	0.51	61.74
40	55.58	4.50	12.90	6.45		0.57	63.82
80	53.67	3.90	13.10	6.55		0.59	64.92
120	50.60	3.50	13.50	6.75		0.63	66.65
160	49.90	3.30	13.70	6.85		0.64	67.03
200	49.80	3.20	13.80	6.90		0.65	67.09
240	43.45	3.00	14.00	7.00		0.73	70.37
280	40.38	2.60	14.30	7.15		0.76	71.83
320	38.99	2.50	14.70	7.35		0.78	72.46
360	36.99	2.50	14.80	7.40		0.80	73.33
400	35.54	2.50	14.90	7.45		0.81	73.94
440	34.70	2.50	15.00	7.50		0.82	74.29
480	34.51	2.20	15.50	7.75		0.82	74.37
520	34.00	2.10	14.10	7.05		0.83	74.57
560	33.80	2.10	14.90	7.45		0.83	74.65
600	33.69	1.80	15.00	7.50		0.83	74.69
640	30.00	2.10	15.60	7.80		0.87	76.08
680	28.30	2.00	16.00	8.00		0.88	76.67
720	23.96	1.70	16.20	8.10		0.91	78.03
760	22.48	1.90	15.60	7.80		0.92	78.44
800	22.00	1.80	15.20	7.60		0.93	78.57
840	21.16	1.50	15.50	7.75		0.93	78.79
880	21.04	1.60	15.40	7.70		0.93	78.82
920	19.44	2.10	15.50	7.75		0.94	79.22
960	14.04	1.50	15.90	7.95		0.97	80.32
1000	8.80	0.70	15.20	7.60		0.99	81.14
1060	1.00	0.60	15.10	7.70		1.00	81.53

## 2 Experimental Design, Materials and Methods

### 2.1 Materials

**Fig. 1:**
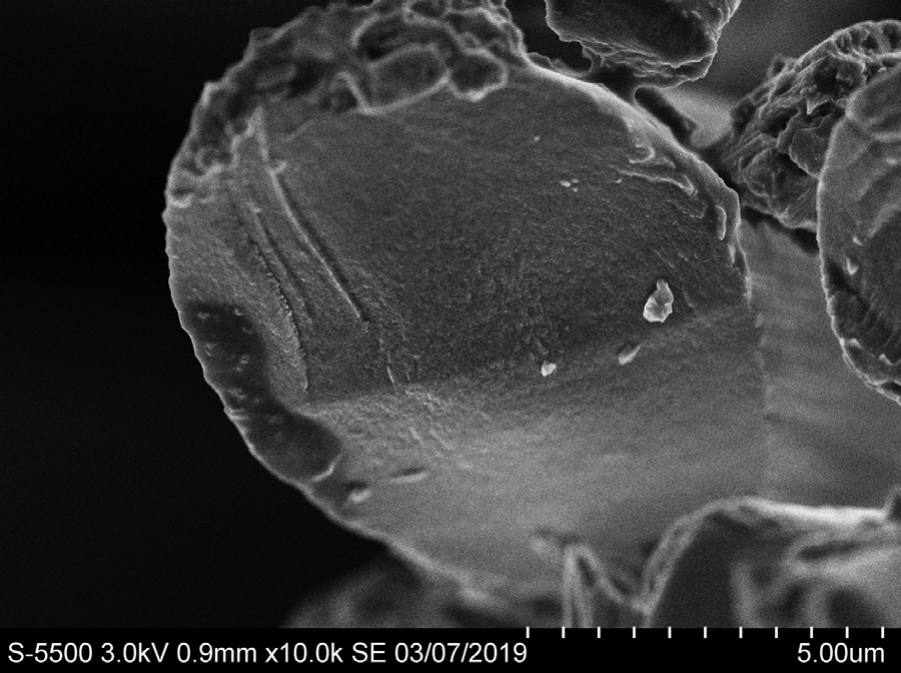
Image of carbon fibers in a scanning electron microscope S-5500.

The object of the study was carbon fiber of Torayca T 300 3 K brand (Japan) with a density 1.76 g/cm^3^, tensile strength 3530 MPa and tensile modulus 230 GPa. Using an ultra-high resolution scanning electron microscope S-5500 (Hitachi, Japan, 2009) made it possible to demonstrate that the carbon fiber is constituted by filaments (Fig. 1). According to the manufacturer's website (https://www.toraycma.com/page.php?id=661), the average diameter of a filament is 7 μm.

**Fig. 2:**
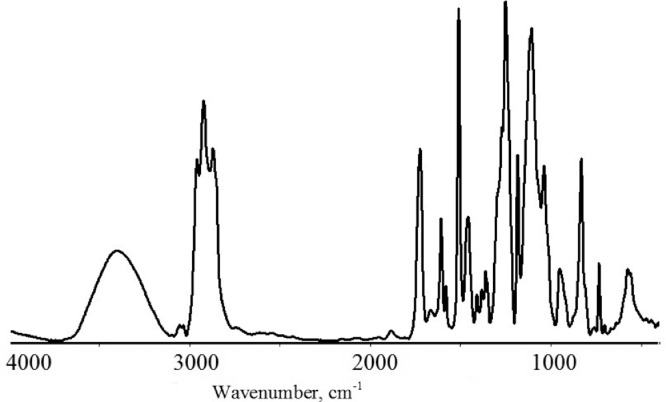
The FT-IR spectrum of the toluene extract of carbon fibers.

### 2.2 Methods

#### 2.2.1 Methods of removing the coating agent from the surface of carbon fiber

To identify the surface properties of carbon fibers the coating agent was extracted with toluene. For this purpose, a glass cell with the testing sample of 2 g. finely chopped carbon fibers was filled with 200 ml of toluene and kept for 48 h at 23 °C. After that, it was treated with ultrasound at a frequency of 22 Hz for 10 min and then kept under the same conditions at 296 °K for 12 h. Following that, the sample was filtrated and used for further experiments.

#### 2.2.2 FT-IR spectroscopy of the coating agent

The carbon fiber extract was analyzed with the Nicolet iS10 FT-IR spectrometer and the Smart iTX™ Accessory from Thermo Scientific (USA) with the diamond attenuated total reflectance method (ATR). The extract was identified as a composition based on bisphenol A di-glycidyl ether identical to epoxy resins.

In the IR range (Table 1 https://data.mendeley.com/datasets/729skbm3g5/3), the absorption bands at 1.260, 917, and 830 cm^−1^ correspond to the epoxy ring stretch and bending ranges. The absorption band at 3.500 cm^−1^ corresponds to the stretch of the *v* (О‒Н) hydroxyl group stretch formed as the epoxy ring opens [2]. The *v* (C‒О‒C) absorption bands at 1.036 and 1.100 cm^−1^ represent the primary and secondary groups. The spectroscopic study results are presented as absorbencies for each peak value.

#### 2.2.3 Thermal gravimetric analysis of carbon fiber

**Fig. 3:**
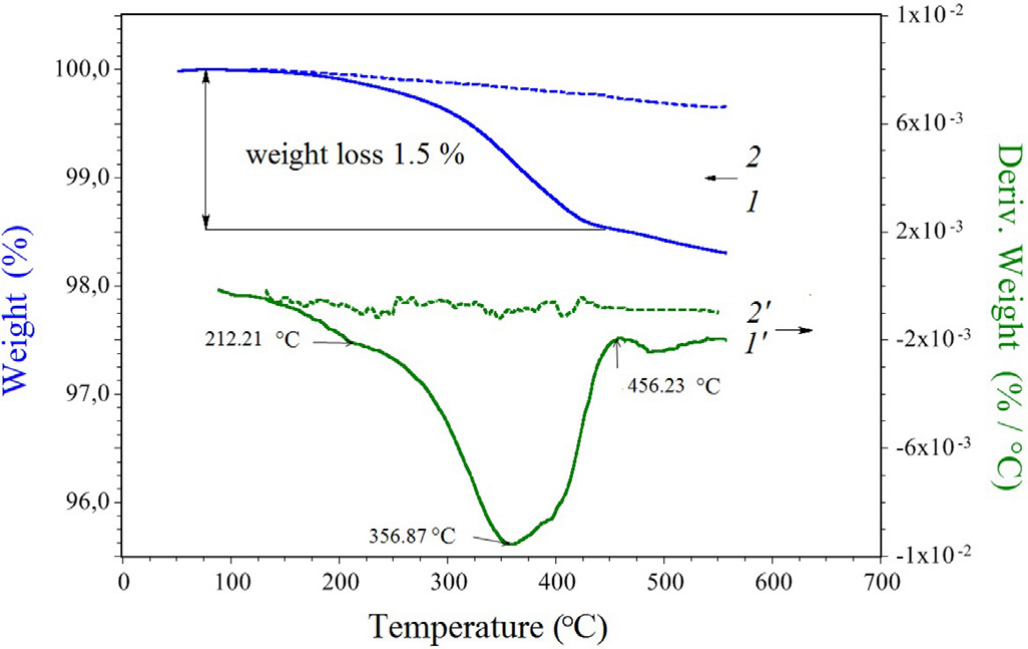
Mass loss and its first derivative vs. temperature before (1) and after (2) thermal treatment of the filaments.

The coating agent was removed from the carbon fibers by bakeout in the air with a muffle furnace at 410 °C for 10 min. The study of the extracted coating agent thermal decomposition with the TGA 55 thermo gravimetric analyzer (TA Instruments, USA) showed that this is the most efficient bake out temperature (Fig. 3).

A supplementary file (with two Excel spreadsheets: Table 2, Table 3 https://data.mendeley.com/datasets/729skbm3g5/3) contains the detailed data.

#### 2.2.4 The change in the capillary ascent height

**Fig. 4:**
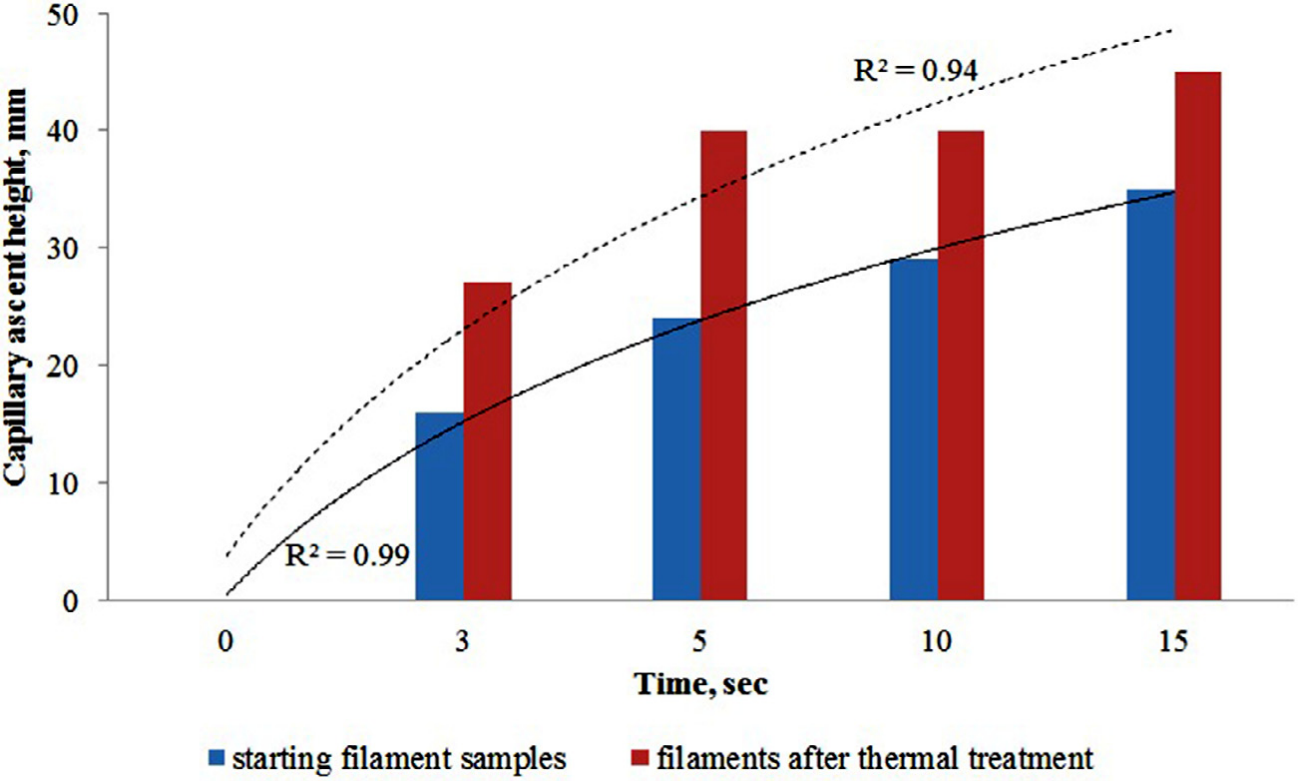
Kinetic curves of wetting in the starting filament samples and filaments after the thermal treatment.

To estimate the effects of the coupling agent on the wetting processes, we analyzed the capillary rise before and after heat treatment (Fig. 4).

The coating agent was removed from the carbon fibers by bake out in the air with a muffle furnace at 410 °C for 10 min (refer to 2.2.3).

The change in the height of the capillary rise is described by logarithmic equations (Fig. 4).

$$y_{1}=21.3\;\ln\left(x\right)+0.4052$$

$$y_{2}=27.853\ln\left(x\right)+3.7308$$

#### 2.2.5 Determination of the contact angle

**Fig. 5:**
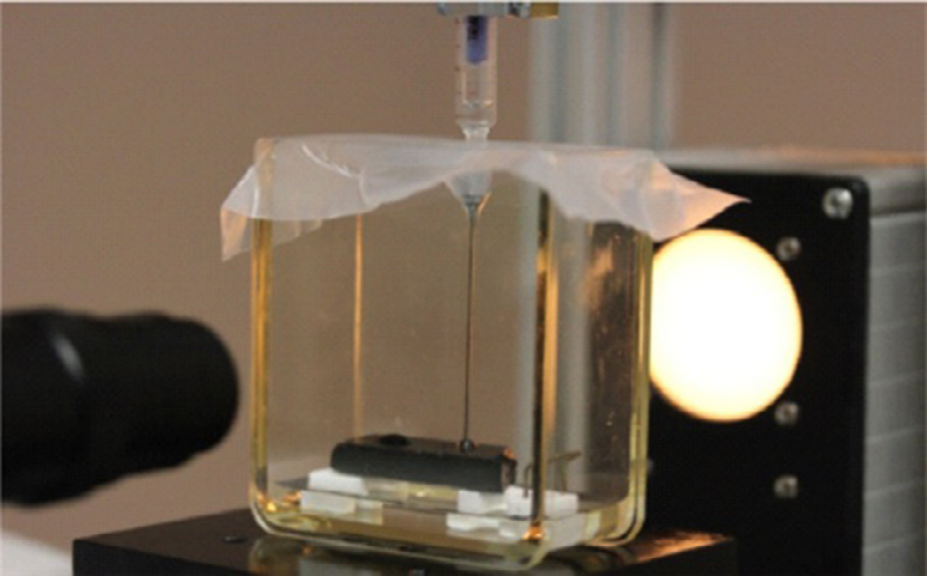
Experimental cell for contact angle measurements.

For measuring the contact angle of the polymeric binders before and after the thermal treatment we analyzed the “sessile drop” shape on the carbon fiber substrate using the sessile-drop technique proposed by Drelich Ja. [3]. The instrument is shown in Fig. 5.

The instrument consists of a video camera, a substrate and a tightly attached to a rack syringe with a prepared binder mixture. A polymeric binder was applied to the fiber, and then the drop profile was recorded with a digital camera.

The footage was loaded into a video editor VirtualDub, licensed GNU General Public License (GPL). Slow motion replay allowed capturing the moment when the drop stopped. The drop parameters such as drop size and the diameter at the point of contact with the fabric sheet were further measured with a GIMP2.0 graphical editor (free software license from GNU General Public License). The obtained values were used to calculate the tangent of the contact angle.

In this study we used the method of determining the coefficient of surface tension and contact angle, including the measurement of geometric characteristics of liquid droplets and their comparison with the numerical solution of differential equations of equilibrium. This method implies that the droplet is shaped on a horizontal solid surface; the measured geometrical characteristics are the height of the peak drops and radius of the spot contact with a solid surface. Adhesion tension is a function of contact angle (between a solid and a liquid), surface tension, and the interfacial tension between the solid and liquid. The Young–Dupre equation exemplifies this relation [4]. The experimental data for the equation are given in Table 5.

Wa=σ(1+cosθ)

## CRediT Author Statement

**Svetlana Yurievna Voronina:** Conceptualization, Methodology, Formal analysis, Writing - original draft, Writing - review & editing, Visualization; **Taisia Alexandrovna Shalygina:** Investigation, Formal analysis, Writing - original draft; **Vasilii Dmitrievich Voronchikhin:** Conceptualization, Formal analysis, Methodology, Writing - original draft; **Anton Yurievich Vlasov:** Conceptualization, Methodology, Writing - original draft; **Anatoly Nikolayevich Ovchinnikov:** Investigation; **Nina Nikolayevna Grotskaya:** Writing - original draft.

## Declaration of Competing Interest

The authors declare that they have no known competing financial interests or personal relationships which have, or could be perceived to have, influenced the work reported in this article.
